# Role of long noncoding RNA KCNQ1 overlapping transcript 1/microRNA-124-3p/BCL-2-like 11 axis in hydrogen peroxide (H_2_O_2_)-stimulated human lens epithelial cells

**DOI:** 10.1080/21655979.2022.2032966

**Published:** 2022-02-16

**Authors:** Yue Xu, Yanhua Zheng, Pincheng Shen, Liping Zhou

**Affiliations:** Department of Ophthalmology, Xiangyang Central Hospital, Affiliated Hospital of Hubei University of Arts and Science, Xiangyang, China

**Keywords:** LncRNA KCNQ1OT1, oxidative damage, miR-124-3p/BCL2L11 axis, age-related cataract

## Abstract

Age-related cataract (ARC) is one of the most common causes of vision loss in aging people. This research analyzed the functions and mechanism of long noncoding RNA KCNQ1 overlapping transcript 1 (KCNQ1OT1) in hydrogen peroxide (H_2_O_2_)-stimulated human lens epithelial cells (SRA01/04 cells) in ARC. SRA01/04 cells were stimulated with 200 µM H_2_O_2_ to establish oxidative damage in the ARC model. A MTT (3-(4,5-dimethylthiazol-2-yl)-2,5-diphenyltetrazolium bromide) assay and flow cytometry analysis were conducted to evaluate cell growth and apoptosis. The relevance between KCNQ1OT1 and microRNA (miR)-124-3p or miR-124-3p and BCL-2-like 11 (BCL2L11) was measured through Starbase and a dual luciferase reporter gene assay. The levels of KCNQ1OT1 and miR-124-3p were assessed via quantitative real-time polymerase chain reaction (qRT-PCR). We observed that KCNQ1OT1 was over-expressed and miR-124-3p was low-expressed in H_2_O_2_-stimulated SRA01/04 cells. KCNQ1OT1 interacted with miR-124-3p and negatively mediated its levels. In addition, KCNQ1OT1-siRNA reversed the effects of H_2_O_2_ on SRA01/04 cells, evidenced by enhanced cell viability, inhibited apoptotic cells, promoted Bcl-2 expression, and reduced Bax levels. Nevertheless, these observations were inverted after miR-124-3p inhibitor treatment. Likewise, miR-124-3p mimic had a protective effect on H_2_O_2_-stimulated SRA01/04 cells. Our data suggested that BCL2L11 targeted miR-124-3p directly. In summary, the data indicated that lncRNA KCNQ1OT1 down-regulation protected SRA01/04 cells from oxidative stress stimulated damage via the miR-124-3p/BCL2L11 pathway.

## GRAPHICAL ABSTRACT

**Figure:**
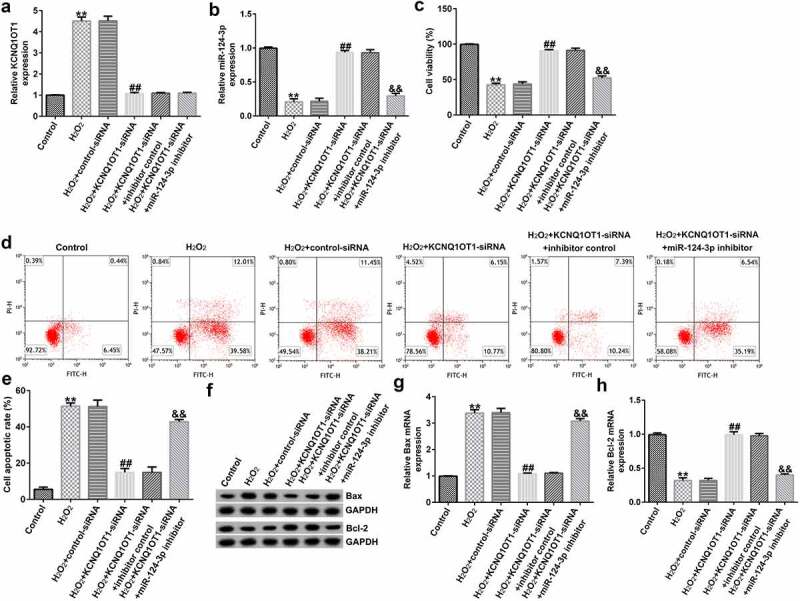

## Introduction

Age-related cataract (ARC), considered as senile cataract, is the most common type of cataract among middle-aged and older adults over 50 years old [1,2]. The incidence rate, related with the degeneration of slow metabolism, increases with age. However, most cases are slow and do not affect vision. The diagnosis of age-related cataract, which affects vision due to lens clouding, is of clinical significance [3]. Although surgery is the most effective treatment method, it also includes complications, which may inevitably lead to irreversible blindness [4,5]. Therefore, further research on the pathogenesis of cataract and new treatment methods is the key to improving its therapeutic effect.

Oxidative stress (OS), an imbalance between oxidation and antioxidation in the body, tends to oxidize cells and leads to neutrophil inflammatory infiltration and the production of extensive oxidative intermediates [6,7]. OS, a side effect of free radicals in an organism, is a supposed key factor which results in aging and pathema [8]. It is associated with the mechanisms of cataract, which are mainly caused by oxygen free radicals induced lens epithelial cells injury, change in protein conformation, and protein cross-linking and accumulation [9]. This stress can cause lens epithelial cells (LECs) apoptosis, leading to cataract, the common cellular basis of ARC. Nevertheless, the concrete mechanisms of oxidative stress-induced LECs apoptosis and ARC are still unclear.

Long noncoding RNA (LncRNA), a kind of ribonucleic acid (RNA) transcript, containing more than 200 nucleotides yet lacks protein coding potential [10]. It is associated with gene regulation and maintaining genome stability, and plays a significant role in cell growth, migration, invasion, apoptosis, and differentiation [11]. According to recent studies, lncRNA expression is closely related with a variety of tumors, which regulates genes expressions and associates with abnormal proliferation and chemotherapy resistance. Yi et al. suggested that lncRNA KCNQ1OT1 accelerated the chemoresistance of oxaliplatin in colorectal carcinoma through the miR-34a/autophagy related 4B pathway [12]. Meanwhile, Wang et al. reported that KCNQ1OT1 accelerated gastric cancer development via miR-4319/DNA damage regulated autophagy modulator 2 axis [13]. However, the function of KCNQ1OT1 in ARC remains unclear, and its mechanism requires further research.

microRNAs (miRNAs), a group of endogenous, small non-coding RNAs, which regulate gene expression post-transcriptionally by binding with 3’-untranslated regions of their target mRNAs [14,15]. Recent research has indicated that miRNAs play an important role in the pathogenesis of cataracts [16–18]. miR-124-3p has been proved to be down-regulated in the cataractous lens samples [19]. Several studies have shown that miR-124-3p plays an important role in the regulation of oxidative stress and inflammatory responses [20,21]. These researches suggested that miR-124-3p may participates in ARC via regulating oxidative stress. Excitingly, bioinformatics analysis revealed the existence of binding sites for KCNQ1OT1 and miR-124-3p.

In the present study, we hypothesized that KCNQ1OT1 plays a role in ARC development through regulating oxidative stress via miR-124-3p. Therefore, Therefore, our study aimed to reveal the underlying functions of KCNQ1OT1 in SRA01/04 cells after oxidative stress and explain its latent mechanisms.

## Materials and methods

### Specimens

20 cases anterior lens capsules were collected from 20 ARC patients (10 male, 10 female; age 52–74 years old) undergoing phacoemulsification surgery at the Xiangyang Central Hospital, Affiliated Hospital of Hubei University of Arts and Science. Besides, another 20 anterior lens capsules of transparent lens (10 male, 10 female; age 55–71 years old) obtained from the Xiangyang Central Hospital, Affiliated Hospital of Hubei University of Arts and Science were served as the Healthy control. All specimens were immediately stored in liquid nitrogen at the time of collection. This study was approved by the Ethics Committee of the Xiangyang Central Hospital, Affiliated Hospital of Hubei University of Arts and Science, and signed informed consent was obtained from each patient.

### Cell cultivation and oxidative stress model establishment

The SRA01/04 cells were obtained from the American Type Culture Collection (ATCC), cultivated in Dulbecco’s modified easy medium (DMEM), which contained 10% FBS (Invitrogen), 1% penicillin, and streptomycin, and maintained at 37°C with 5% CO2 in an incubator. The cells were stimulated with 200 µM H_2_O_2_ for 24 h to conduct the oxidative stress model *in vitro* [22].

### Dual-luciferase reporter assay [23]

TargetScan (StarBase, Microrna) was used to investigate the latent target genes of KCNQ1OT1. We built the reporter vector KCNQ1OT1 wild-type (KCNQ1OT1-WT) or KCNQ1OT1 mutated-type (KCNQ1OT1-MUT) 3’-UTR luciferase reporter gene plasmids using genomic PCR and pMIR vectors (Ambion, USA). Next, miR-124-3p wild-type or mutant portion bound to miR-124-3p, mimic or mimic control, were added into 293 cells using Lipofectamine 2000 (Invitrogen) according to the instructions. Luciferase activity was assessed via a dual-luciferase reporter assay (Promega).

### qRT-PCR analysis

After treatment, KCNQ1OT1 and miR-124-3p levels were examined using qRT-PCR. The separation of RNA from SRA01/04 was conducted by TRIzol reagent (TaKara, Shiga, Japan) according to the instructions. Next, the total RNA was reversed to cDNA in accordance with the instructions of the PrimeScript RT Reagent Kit (TaKaRa, China). Furthermore, qRT-PCR analysis was performed by the SYBR PrimeScript RT-PCR Kit (Vazyme, Nanjing, Jiangsu) with ABI PRISM 7900 sequence detection system (Vazyme, Nanjing, Jiangsu). The target gene expressions were assessed by 2^−ΔΔCt^ formula [24].

### Western blot analysis [25]

The total proteins were harvested from the SRA01/04 cells with a radioimmunoprecipitation (RIPA) lysis buffer (Beyotime). The protein consistencey was evaluated by NanoDrop. The specimens were resolved by 10% sodium dodecyl sulfate-polyacrylamide gel electrophoresis (SDS-PAGE) and diverted to poly(vinylidene fluoride) (PVDF) membrane. After they were treated with 5% nonfat milk for 1 h, the membranes were cultivated in primary antibodies against β-actin, Bcl2, and Bax (1: 1000 dilution) overnight at 4°C. Following that, the membranes were treated with secondary antibodies for 1 h. Finally, the protein signals were detected by the enhanced chemiluminescence (ECL) detection system reagents (Pierce, USA) and quantified using Image J Software.

### Cell transfection

The control-siRNA, KCNQ1OT1-siRNA, inhibitor control, miR-124-3p inhibitor, mimic control or miR-124-3p mimic were synthesized by GenePharma (Shanghai, China) and transfected into the SRA01/04 cells by Lipofectamine™3000 (Invitrogen) according to the instructions. After 48 hours of treatment, RNA was extracted for qRT-PCR analysis, and the Western blot was used to evaluate the protein expression.

### MTT assay [26]

The SRA01/04 cells were cultured in 96-well plates at 37°C. Next, the cells were mixed with 10 μl MTT (5 mg/ml) solution and cultivated for 4 h. The culture medium was removed and 150 ul of dimethyl sulfoxide (DMSO) was added to dissolve the formazan product in darkness for 10 min. Finally, the OD_570nm_ wavelength was measured by a microplate reader (BioTek, USA) after vibration mixing according to the instructions.

### Flow cytometry analysis [27]

After treatment, SRA01/04 cells apoptosis was checked by an annexin V-FITC/PI apoptosis detection kit (BD Bioscience) according to the instructions. Finally, the cells were assessed by the BD FACSCalibur flow cytometer (BD Technologies) and analyzed using FlowJo Analysis software.

### Statistical analysis

All results were expressed as mean ± SD from three independent experiments. A one-way analysis of variance (ANOVA) or Student’s t-test was used to estimate the mean diversity between the groups. Statistical significance was set as *p* < 0.05.

## Results

### miR-124-3p directly interacted with KCNQ1OT1

**Figure 1.**
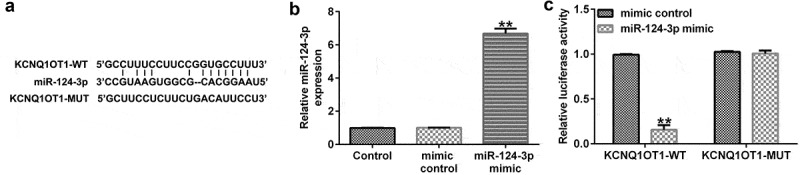
miR-124-3p directly targeted KCNQ1OT1.

Previous reports confirmed that lncRNAs and miRNAs were involved in cataracts. In addition, KCNQ1OT1 was significantly up-regulated in cataracts. Thus, we first investigated the correlation between KCNQ1OT1 and miRNAs using bioinformatics tools (Starbase) which revealed the binding sites between miR-124-3p and KCNQ1OT1 (Figure 1(a)). Furthermore, it was revealed that miR-124-3p mimic significantly enhanced miR-124-3p levels in 293 T cells compared to the mimic control group (Figure 1(b)). Furthermore, the dual-luciferase reporter assay suggested that miR-124-3p mimic significantly reduced KCNQ1OT1-WT luciferase activity, while KCNQ1OT1-MUT luciferase activity had no change (Figure 1(c)), which suggested that miR-124-3p directly interacted with KCNQ1OT1.

(a) The predicted KCNQ1OT1 binding site within miR-124-3p WT or MUT 3’-UTR is displayed. (b) A qRT-PCR analysis of miR-124-3p level in mimic control or miR-124-3p mimic transfected 293 T cells. (c) Luciferase activities were assessed via the dual-luciferase reporter assay system. **p < 0.01 vs. control.

*KCNQ1OT1 was significantly over-expressed and miR-124-3p was low-expressed in H_2_O_2_-stimulated SRA01/04 cells*

**Figure 2.**
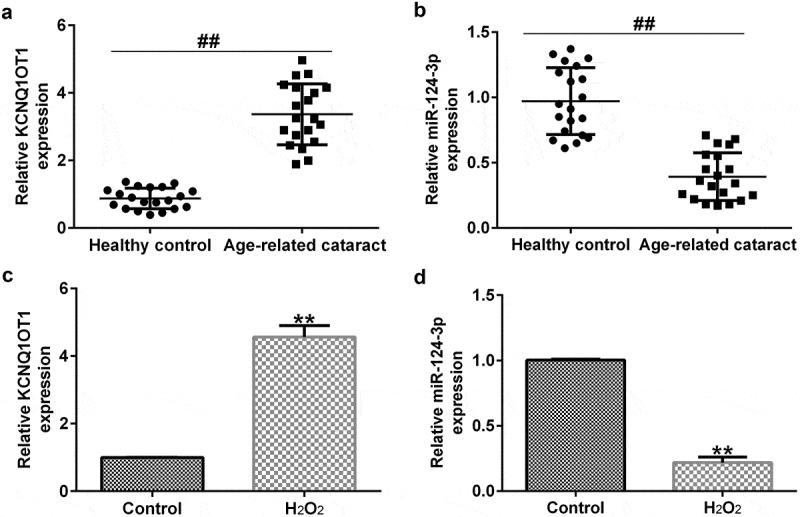
Effects of H_2_O_2_ on miR-124-3p and KCNQ1OT1 level in SRA01/04 cells.

To confirm the expression of miR-124-3p and KCNQ1OT1 in ARC, the level of miR-124-3p and KCNQ1OT1 in anterior lens capsules of ARC patients were determind using RT-qPCR. Results indicated that compared with the healthy control group, KCNQ1OT1 was significantly enhanced, while miR-124-3p was down-regulated in the anterior lens capsules of ARC patients (Figure 2(a,b)). SRA01/04 cells were exposed to 200 µM H_2_O_2_ to generate oxidative stress model *in vitro*. The miR-124-3p and KCNQ1OT1 levels in the H_2_O_2_-induced SRA01/04 cells were assessed via qRT-PCR analysis. As displayed in Figure 2(c,d), KCNQ1OT1 levels were higher in the H_2_O_2_-treated SRA01/04 cells compared to the control group. Our findings demonstrated that miR-124-3p was significantly down-regulated after H_2_O_2_ treatment, which indicated that KCNQ1OT1/miR-124-3p played a regulatory role in H_2_O_2_-stimulated SRA01/04 cells.

The expression of KCNQ1OT1 (a) and miR-124-3p (b) in anterior lens capsules of ARC patients were determind using qRT-PCR. A qRT-PCR analysis of (c) KCNQ1OT1 and (d) miR-124-3p expression in H_2_O_2_-stimulated SRA01/04 cells. ##*p* < 0.01; **p < 0.01 vs. control.

### KCNQ1OT1 negatively regulated miR-124-3p expression in SRA01/04 cells

**Figure 3.**
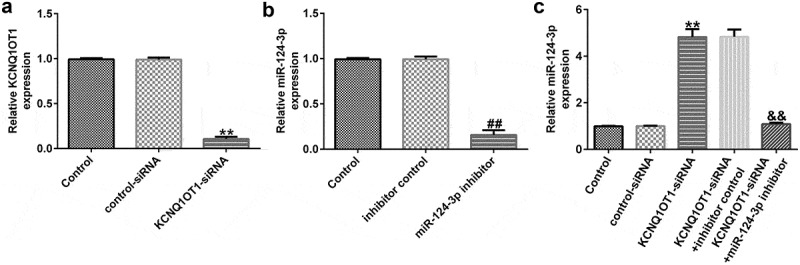
Down-regulation of miR-124-3p abolished the influence of KCNQ1OT1-siRNA on miR-124-3p level in SRA01/04 cells.

To further explore the regulatory functions of KCNQ1OT1 and miR-124-3p in the H_2_O_2_-stimulated SRA01/04 cells, they were exposed to control-siRNA, KCNQ1OT1-siRNA, inhibitor control or miR-124-3p inhibitor. Our data suggested that KCNQ1OT1-siRNA reduced KCNQ1OT1 levels in SRA01/04 cells (Figure 3(a)). Moreover, our findings (as shown in Figure 3(b)) revealed that miR-124-3p was down-regulated after miR-124-3p inhibitor transfection compared to the inhibitor control group. In addition, compared to the control-siRNA group, KCNQ1OT1-siRNA significantly promoted miR-124-3p expression in SRA01/04 cells. However, this promotion was eliminated by miR-124-3p inhibitor (Figure 3(c)), which suggested that KCNQ1OT1 negatively regulated miR-124-3p expression in SRA01/04 cells.

SRA01/04 cells were treated with control-siRNA, KCNQ1OT1-siRNA, inhibitor control, or miR-124-3p inhibitor for 24 h. (a) A qRT-PCR analysis of KCNQ1OT1 in control-siRNA or KCNQ1OT1-siRNA transfected SRA01/04 cells. (b) Detection of miR-124-3p levels in inhibitor control or miR-124-3p inhibitor transfected SRA01/04 cells. (c) miR-124-3p expression in KCNQ1OT1-siRNA+inhibitor control or KCNQ1OT1-siRNA+miR-124-3p inhibitor transfected cells. **p < 0.01 vs. control.

*Down-regulation of miR-124-3p reversed the roles of KCNQ1OT1-siRNA in H_2_O_2_-stimulated oxidative stress in SRA01/04 cells*

**Figure 4.**
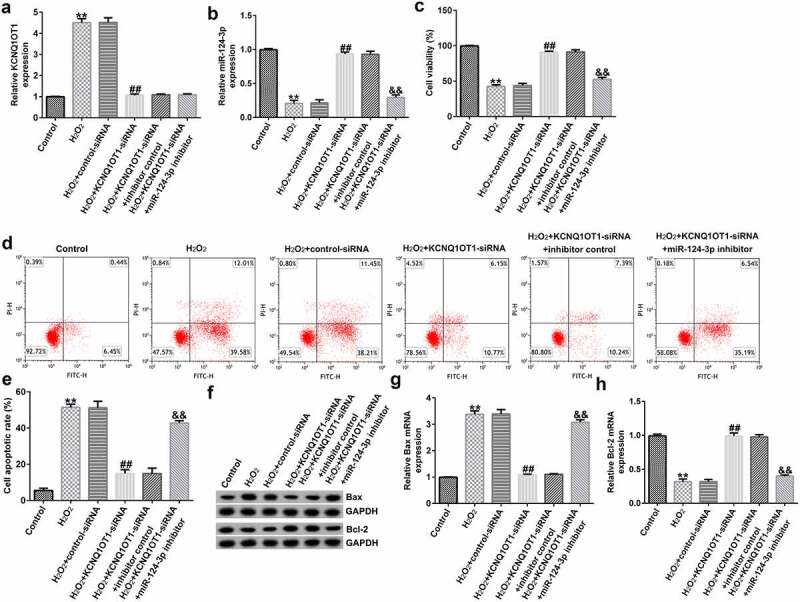
Down-regulation of miR-124-3p abolished the influence of KCNQ1OT1-siRNA on cells growth and apoptosis in H_2_O_2_-stimulated SRA01/04 cells.

To further study the roles of miR-124-3p in H_2_O_2_-stimulated SRA01/04 cells, the SRA01/04 cells were induced by control-siRNA, KCNQ1OT1-siRNA, inhibitor control or miR-124-3p inhibitor, followed by 200 µM H_2_O_2_. Next, we conducted a qRT-PCR analysis to evaluate the miR-124-3p and KCNQ1OT1 levels in different groups. We found that the level of KCNQ1OT1 was higher and miR-124-3p was lower in H_2_O_2_-stimulated SRA01/04 cells than that in control group (Figure 4(a,b)). Compared to H_2_O_2_+ control-siRNA group, the expression of KCNQ1OT1 was reduced and miR-124-3p expression was fortified in KCNQ1OT1-siRNA transfected cells (Figure 4(a,b)), while the increased miR-124-3p level which resulted from KCNQ1OT1-siRNA transfection was reversed by down-regulating miR-124-3p (Figure 4(a,b)). Further, the MTT and flow cytometry assay suggested that KCNQ1OT1-siRNA promoted growth in H_2_O_2_-stimulated SRA01/04 cells, and this increase was eliminated by down-regulating miR-124-3p (Figure 4(c)). In addition, KCNQ1OT1-siRNA stimulation resulted in reduced apoptotic cells, increased Bcl-2 expression and reduced Bax level. Furthermore, these data were eliminated by miR-124-3p inhibitor, which suggested that KCNQ1OT1 relieved H_2_O_2_-stimulated oxidative stress via regulating miR-124-3p (Figure 4(d-h)).

After transfection and treatment, SRA01/04 cells were divided into six groups: Control, H_2_O_2_,H_2_O_2_+ control-siRNA,H_2_O_2_+ KCNQ1OT1-siRNA,H_2_O_2_+ KCNQ1OT1-siRNA+inhibitor control, or H_2_O_2_+ KCNQ1OT1-siRNA+miR-124-3p inhibitor group. (a-b) A qRT-PCR analysis of KCNQ1OT1 or miR-124-3p in six groups. (c) The cells viability was evaluated using a MTT analysis. (d) SRA01/04 cells apoptosis was calculated using flow cytometry. (e) Quantitation of apoptotic cells. (f) Western blot analysis of Bcl-2 and Bax expression. (g and h) A qRT-PCR analysis of Bax and Bcl-2. **p < 0.01 vs. control.

### miR-124-3p relieved H_2_O_2_-stimulated cell injury in SRA01/04 cells

**Figure 5.**
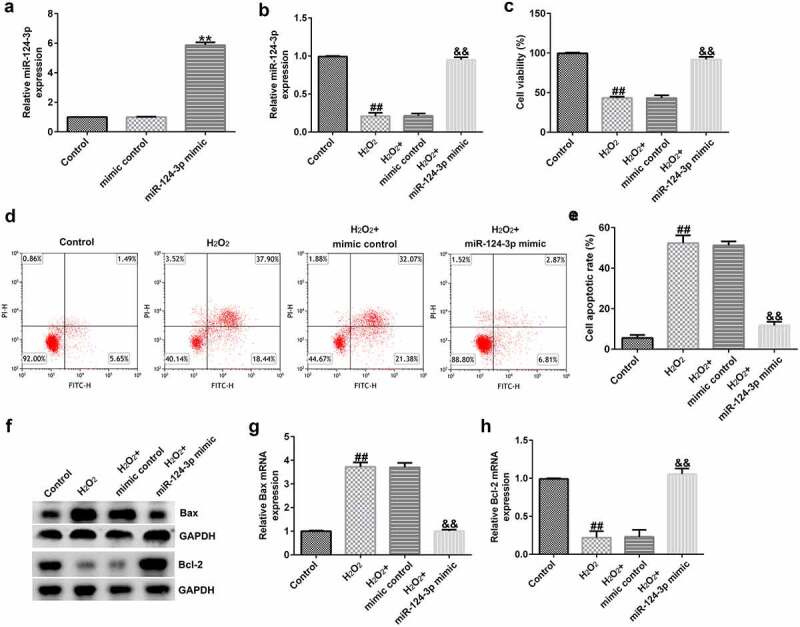
Influence of miR-124-3p on H_2_O_2_-stimulated SRA01/04 cells viability and apoptosis.

To lucubrate the roles of miR-124-3p in H_2_O_2_-stimulated SRA01/04 cells, SRA01/04 cells were induced by mimic control or miR-124-3p mimic and 200 µM H_2_O_2_ for 24 h. Results from a qRT-PCR suggested that up-regulation of miR-124-3p enhanced the messenger RNA (mRNA) level of miR-124-3p in SRA01/04 cells compared to mimic control (Figure 5(a)). The results (as shown in Figure 5(b-e)) confirmed that H_2_O_2_ led to inhibited miR-124-3p expression, depressed cell growth, as well as promoted apoptotic cells. Furthermore, H_2_O_2_ decreased Bcl-2 expression and increased Bax level (Figure 5(f-h)). However, all these findings were reversed by miR-124-3p mimic, which suggested that miR-124-3p alleviated H_2_O_2_-induced cells injury in SRA01/04 cells.

Cells were transfected with mimic control or miR-124-3p mimic and stimulated with 200 µM H_2_O_2_ for 24 h. (a-b) Detection of miR-124-3p levels. (c) Cells growth and (d) cell apoptosis were calculated via a MTT and flow cytometry analysis. (e) Quantitative analysis of the apoptotic cells. (f) Determination of Bax and Bcl-2 expression using Western blot. (G and H) A qRT-PCR analysis of Bax and Bcl-2. **p < 0.01 vs. control.

### BCL2L11 directly targeted miR-124-3p

**Figure 6.**
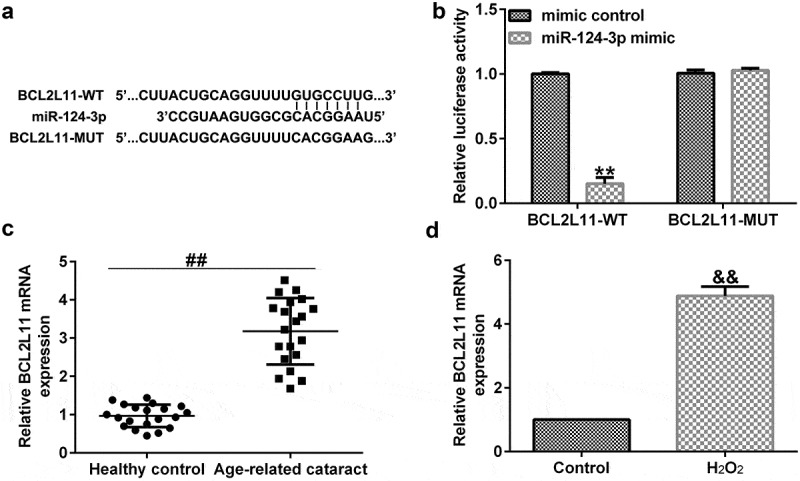
BCL2L11 directly targeted miR-124-3p.

As we had already explored the relevance between KCNQ1OT1 and miR-124-3p, we explained the latent mechanisms of miR-124-3p in SRA01/04 cells. TargetScan analysis revealed that BCL2L11 was a candidate target of miR-124-3p (Figure 6(a)). Further, the dual luciferase reporter gene system verified that BCL2L11 was a sponge for miR-124-3p (Figure 6(b)). Moreover, we found that compared with the healthy control group, BCL2L11 was significantly enhanced in the anterior lens capsules of ARC patients (Figure 6(c)). And BCL2L11 level was higher in the H_2_O_2_-treated SRA01/04 cells compared to the control group (Figure 6(d)). All the abovementioned findings revealed that down-regulation of lncRNA KCNQ1OT1 protected LECs from oxidative stress stimulated apoptosis through mediating the miR-124-3p/BCL2L11 pathway in ARC.

(a) A schematic of BCL2L11 binding site in miR-124-3p 3’-UTR. (b) Luciferase activities were checked via the dual-luciferase reporter assay system. The expression of BCL2L11 in (c) anterior lens capsules of ARC patients and in H_2_O_2_-stimulated SRA01/04 cells were determind using qRT-PCR. **p < 0.01 vs. mimic control; ##*p* < 0.01; &&*p* < 0.01 vs. control.

## Discussion

The novel breakthroughs of this research included: i) Down-regulation of lncRNA KCNQ1OT1 alleviated oxidative stress induced injury in SRA01/04 cells; ii) the protective effects of KCNQ1OT1 on H_2_O_2_-stimulated cells were regulated through the miR-124-3p/BCL2L11 pathway. To our knowledge, we are the first study to explained the functions of KCNQ1OT1 on H_2_O_2_-stimulated cells apoptosis and investigate the latent underlying mechanisms.

There are three major forms of ARC, which include the cortical cataract, nuclear cataract, and posterior subcystic cataract [28]. Regardless of how they differ, they are associated with abnormal growth or apoptotic cell death in the lens. Recently, studies on the pathogenesis of cataract found that LECs apoptosis was closely associated with the pathogenesis of cataract, which has attracted widespread attention [29,30]. Li et al. suggested that microRNA-15a regulated LECs apoptosis and viability by targeting Bcl-2 and E2F3 in ARC [31]. Furthermore, Lu et al. suggested that miR-211 accelerated LECs apoptosis by targeting SIRT1 in ARC. In addition, previous studies have shown that oxidative stress was identified as one of the prime reasons for cataract [32]. Exposure of lens epithelial cells to ultraviolet light, calcium ions, or H_2_O_2_ may lead to an increase in the abundance of apoptotic proteins, thereby inducing cell death, which is a familiar cellular basis for ARC [33]. In our research, SRA01/04 cells were stimulated with 200 µM H_2_O_2_ to conduct the ARC model *in vitro.*

Various lncRNAs, which included lncRNA PLCD3-OT1 and lncRNA H19, have been evidenced to be associated with ARC [34,35]. LncRNA KCNQ1OT1, has also been evidenced to be related to the advance of multiple diseases, including cancer [11–13] and cataract [36–38]. Jin et al. reported that KCNQ1OT1 silencing significantly inhibited H_2_O_2_-induced SRA01/04 cell pyroptosis, which is the critical step in cataract formation [36]. Liu et al indicated that knockdown of KCNQ1OT1 inhibits cell viability, migration and epithelial-mesenchymal transition in high glucose-treated lens epithelial cells in diabetic cataract [38]. However, the functions of KCNQ1OT1 in ARC have not been fully explained. Based on Starbase and the dual-luciferase reporter analysis, we observed that miR-124-3p was a sponge of KCNQ1OT1. MiR-124-3p was a tumor regulator in many cancers, such as angiotensin II-dependent hypertension [39], gastric cancer [40], and peripheral arterial disease [41]. Besides, miR-124-3p has been revealed to play important roles in the regulation of oxidative stress, which is the critical factor in ARC [20,21]. Nevertheless, miR-124-3p functions in ARC are largely indistinct. Consistent with previous studies [36,37], we further verified that KCNQ1OT1 was remarkably over-expressed in the anterior lens capsules of ARC patients and in H_2_O_2_-stimulated SRA01/04 cells. While miR-124-3p was down-expressed in anterior lens capsules of ARC patients and in H_2_O_2_-stimulated SRA01/04 cells. Therefore, we hypothesized that KCNQ1OT1 participated in ARC through regulating miR-124-3p. And we confirmed that KCNQ1OT1 negatively regulated miR-124-3p expression in SRA01/04 cells.

To better illustrate the KCNQ1OT1/miR-124-3p functions in H_2_O_2_-treated SRA01/04 cells, they were exposed to control-siRNA, KCNQ1OT1-siRNA, inhibitor control, or miR-124-3p inhibitor, and 200 µM H_2_O_2_ for another 24 h. Results from the MTT and flow cytometry revealed that KCNQ1OT1-siRNA promoted SRA01/04 cells viability and decreased apoptotic cells. Further analysis suggested that Bax was down-expressed and Bcl-2 was up-regulated in lens epithelial cells from cataract patients, which were reversed via miR-124-3p inhibitor.

To further explore the roles of miR-124-3p in ARC, the SRA01/04 cells were treated with mimic control, miR-124-3p mimic, or H_2_O_2_ for 24 h. Our data indicated that miR-124-3p mimic enhanced the mRNA level of miR-124-3p in SRA01/04 cells compared to mimic control. Meanwhile, H_2_O_2_ led to inhibited miR-124-3p expression, depressed cell growth, as well as promoted apoptotic cells, decreased Bcl-2 expression, and increased Bax levels. However, all these observations were reversed through miR-124-3p mimic, which indicated that miR-124-3p alleviated H_2_O_2_-induced SRA01/04 cells growth and apoptosis. BCL2L11, a novel target of miR-9, was found to provoke apoptosis [42,43]. Our data further implied that BCL2L11 was a direct target of miR-124-3p, and it was significantly enhanced in the anterior lens capsules of ARC patients and in the H_2_O_2_-treated SRA01/04 cells.

## Conclusion

The finding revealed that down-regulation of lncRNA KCNQ1OT1 protected LECs from oxidative stress stimulated apoptosis via regulating the miR-124-3p/BCL2L11 pathway in age-related cataract. Therefore, it is expected to be a promising avenue for ARC therapy. However, further investigations are required to illustrate the mechanisms of how BCL2L11 expression affects age-related cataract.

## Data Availability

The datasets used and/or analyzed during the current study are available from the corresponding author upon reasonable request.

## Research highlights

- KCNQ1OT1 was significantly over-expressed and miR-124-3p was low-expressed in H_2_O_2_-stimulated SRA01/04 cells;
- Down-regulation of miR-124-3p reversed the roles of KCNQ1OT1-siRNA in H_2_O_2_-stimulated oxidative stress in SRA01/04 cells;
- miR-124-3p relieved H_2_O_2_-stimulated cell injury in SRA01/04 cells.
